# Intramammary Labeling of Epithelial Cell Division

**DOI:** 10.1007/s10911-024-09570-4

**Published:** 2024-10-16

**Authors:** Maia N. Machiela, Russell C. Hovey

**Affiliations:** Department of Animal Science, University of California, Davis One Shields Avenue, Davis, CA 95616-8521 USA

**Keywords:** Mammary gland, Ethynyl deoxyuridine, Bromodeoxyuridine, Sheep

## Abstract

Thymidine analogs such as ethynyl deoxyuridine (EdU) or bromodeoxyuridine (BrdU) can be used to label mitosis of mammary epithelial cells (MEC) and to quantify their proliferation. However, labeling cells in larger animals requires considerable amounts of chemical that can be costly and hazardous. We developed a strategy to infuse EdU into the mammary glands of ewes to directly label mitotic MEC. First, each udder half of nulliparous ewes (*n* = 2) received an intramammary infusion of one of four different concentrations of EdU (0, 0.1, 1.0 or 10 mM) which was compared to BrdU IV (5 mg/kg) 24 h later. Tissues were analyzed by immunofluorescent histochemistry to detect EdU, BrdU, and total MEC. Of the EdU doses tested, 10 mM EdU yielded the greatest labeling index, while a proportion of MEC were labeled by both EdU and BrdU. We next sought to establish whether intramammary labeling could detect the induction of mitosis after exposure to exogenous estrogen and progesterone (E + P). We first infused EdU (10 mM) into the right udder half of ewes (*n* = 6) at t 0, followed by thymidine (100 mM) 24 h later to prevent further labeling. Three ewes were then administered E + P for 5 d, while *n* = 3 ewes served as controls. On d 5, EdU was infused into the left udder half of all mammary glands alongside BrdU IV (5 mg/kg). By the time of necropsy 24 h later an average MEC labeling index of 2.9% resulted from EdU delivered at t 0. In the left half of the udder on d 5, CON glands had a final EdU labeling index of 3.4% while glands exposed to E + P had a labeling index of 4.6% (*p* = 0.05). The corresponding degree of labeling with BrdU was 5.6% in CON glands, and 12% following E + P (*p* < 0.001). Our findings reveal that intramammary labeling is an efficient and cost-effective method for single- and dual-labeling of cell division in the mammary glands.

**Supplementary Information:**

The online version contains supplementary material available at 10.1007/s10911-024-09570-4.

## Introduction

Growth of the mammary epithelium during postnatal development is critical for the coordinated establishment of the ductal system and organization of mammary epithelial cells (MEC) into functional alveoli. The importance of this cumulative growth is highlighted by the fact that across a broad range of species, the milk yield capacity of any given gland is largely proportional to its number of MEC [1–3].

Various methods have been used to measure changes in mammary growth across an array of experimental and practical settings. Proximate analyses of homogenized tissue extracts have been used to quantify total DNA or RNA as an indicator of cell number within the tissue, or the dried and fat-free parenchyma [4]. In the case of larger species, such as in sheep, pigs or cows, these approaches have been combined with the trimming of extraparenchymal tissues such as the surrounding mammary fat pad [5–7].

However, these methods do not provide any information about tissue structure or spatial patterns of growth. Histological approaches address these issues and afford the ability to immunolocalize and quantify the distribution of nuclear antigens such as Ki-67, proliferating cell nuclear antigen, cluster of differentiation 1, and phosphorylated-histone 3 [3, 8, 9]. Alternatively, the incorporation of thymidine analogs such as [^3^H]-thymidine, bromodeoxyuridine (BrdU), or more recently, ethynyl deoxyuridine (EdU), into replicating DNA can provide spatial data while also quantifying the rate of cellular proliferation across time [10–13], length and turnover of the cell cycle [3, 14], and populations of quiescent and proliferative cells that possibly indicate a stem/progenitor state [15–17].

Typical approaches for labeling MEC with these analogs is either via systemic delivery [3, 18] or in mammary explants collected at necropsy [19]. As opposed to BrdU which requires denaturation of labeled DNA prior to immunohistochemical detection [20], the detection of EdU utilizes a simple and rapid copper-catalyzed “click” reaction [21]. The use of EdU has gained popularity, although it has been used mostly in rodents and in vitro [18, 22, 23], perhaps because of its relatively high cost and the need to treat it as a potential carcinogen. Indeed, given the relatively large size of livestock, systemic labeling requires considerable amounts of nucleotide which can be costly and create additional safety concerns.

Given the utility of EdU for marking cell division, we saw an opportunity to infuse EdU directly into the mammary glands to directly label MEC division. The mammary gland is a closed subcutaneous exocrine system, where any molecule infused into the gland remains concentrated. Indeed, the intramammary route has been used to investigate a variety of local responses by MEC, including to determine the effect of growth factors or hormones on milk yield in dairy cows [24–26], which also may benefit from the intramammary delivery of antibiotics to treat mastitis [27]. In a similar way, pathogens have been instilled into the mammary glands to induce and subsequently characterize the inflammatory response to mastitis [28, 29], while tumor cells have been injected into the ductal system of mice as a model for ductal carcinoma in situ [30].

Direct labeling of dividing MEC would offer a convenient and effective means to trace MEC across time, potentially in combination with other nuclear antigens or nucleotide analogs, while reducing the associated costs and risks, especially in larger species. To this end, we tested and refined a method in sheep to directly label MEC using intramammary infusions of EdU alongside systemic delivery of BrdU. Our results show that intramammary delivery of EdU leads to a dose-dependent labeling of MEC in the mammary glands of nulliparous ewes and can be used to detect a hormone-induced increase in proliferation. The co-administration of BrdU systemically also affords the opportunity to undertake dual-labeling studies for the monitoring of cellular kinetics over time. This method provides a safer, cost-effective, and convenient method for studying cell division events in the mammary glands that has potential utility across a range of species and experimental paradigms.

## Methods

All protocols were approved by the UC Davis Institutional Animal Care and Use Committee. Nulliparous Suffolk ewes from the UC Davis flock were housed in 4 m x 8 m pens where they had *ad libitum* access to alfalfa hay and water. All solutions for intramammary or IV delivery were dissolved in sterile 0.9% sodium chloride and filtered at 0.22 μm. All intramammary infusions were through 22 G 2.5 cm catheters (BD, Franklin Lakes, NJ), and all infusates were 0.5 mL. After each intramammary infusion, VetBond (3 M, St. Paul, MN) was applied to seal each teat sphincter. Systemic infusion of BrdU (Roche, Indianapolis, IN; 5 mg/kg BW) was via an 18 G 4.75 cm catheter (BD, Franklin Lakes, NJ). In Experiment 1, ewes were sedated with xylazine (0.1 mg/kg, IM), while in Experiment 2 they were sedated with midazolam (0.4 g/ kg, IM). Animals were euthanized via captive bolt followed by exsanguination and all carcasses were incinerated.

### Experiment 1: Intramammary Dose

**Fig. 1:**
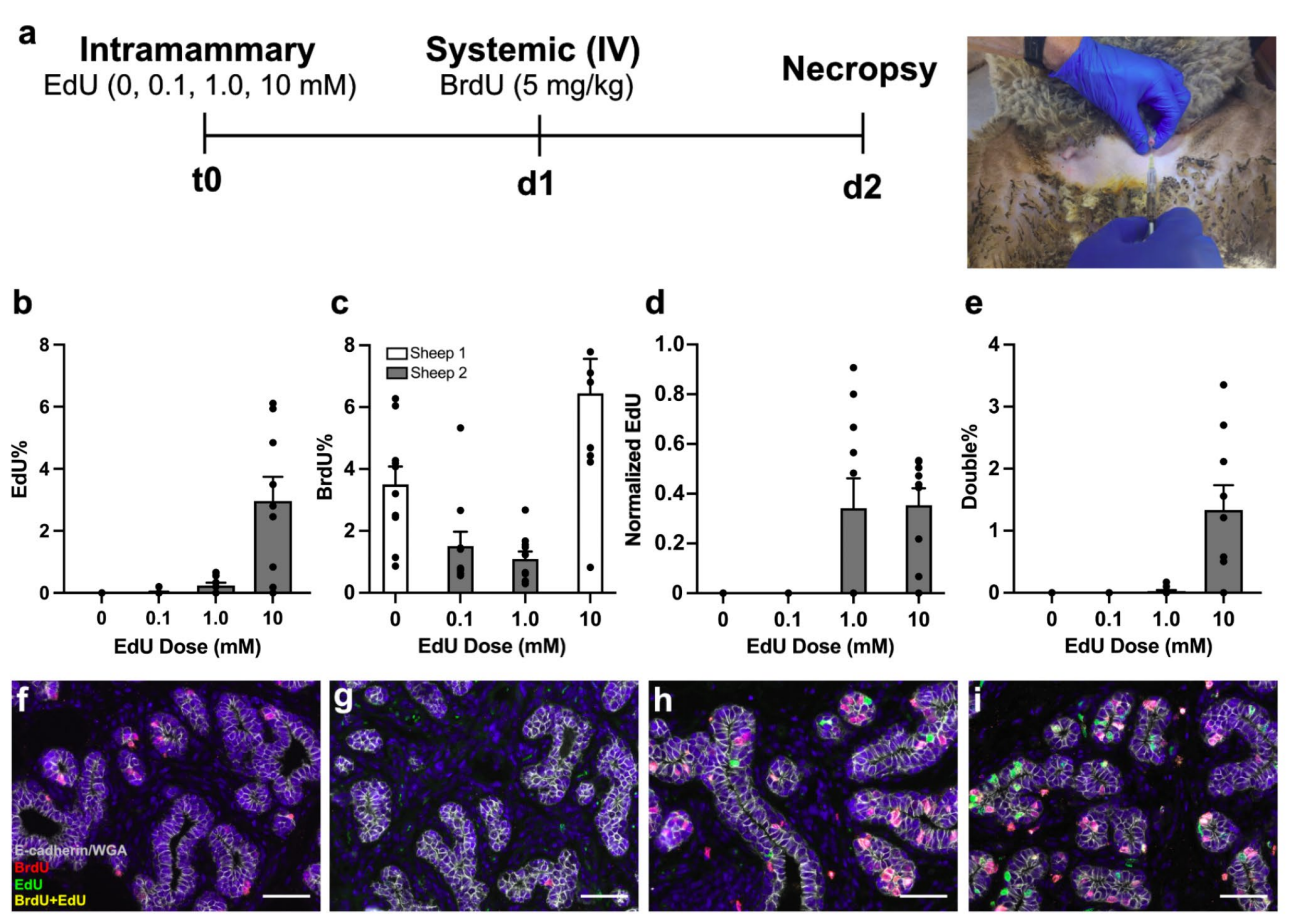
Intramammary label dosing. (**a**) At t 0, four different concentrations of ethynyl deoxyuridine (EdU; 0, 0.1, 1.0 or 10 mM) were infused into separate mammary glands of nulliparous ewes (*n* = 2). A single dose of bromodeoxyuridine (BrdU, IV, 5 mg/kg) was delivered 24 h later on d 1, and mammary tissues were collected at necropsy on d 2. Image panel depicts a catheter inserted through the teat sphincter for intramammary delivery of EdU at t 0. (**b**) Proportion of mammary epithelial cells (MEC) labeled in response to different concentrations of EdU delivered into the mammary glands. (**c**) The proportion of MEC in the separate udder halves labeled by BrdU differed between sheep 1 and sheep 2 (*p* < 0.001). (**d**) Labeling index for EdU expressed per field, normalized to the corresponding BrdU labeling incidence in the same field. Data are normalized averages across all fields. (**e**) Incidence of dual-labeled MEC following intramammary EdU and systemic BrdU. (**f**–**i**) Representative images of epithelial structures in ewes labeled with either 0, 0.1, 1.0, or 10 mM EdU, respectively, at t 0, followed by systemic BrdU that was delivered on d 1. Specimens were imaged to reveal MEC that were positive for EdU (green), BrdU (red), E-cadherin and WGA (grey), and DAPI (blue). Scale bars are 50 μm

Using *n* = 2 ewes (12 mo old), we first evaluated MEC labeling in response to four different concentrations of EdU (0 mM, 0.1 mM, 1.0 mM, or 10 mM) infused via the teat (Fig. 1a). The different concentrations were randomly assigned to the four udder halves. After 24 h, BrdU was administered IV to compare the intramammary and systemic routes. Necropsy occurred 24 h post-BrdU infusion, and 9 or 10 tissue specimens were collected from each bisected udder half, with sampling sites spanning all regions of the visible parenchyma (Supplementary Fig. 1). Tissue specimens were fixed within 15 min of necropsy.

### Experiment 2: Hormone-Induced Mammary Growth and Temporality

**Fig. 2:**
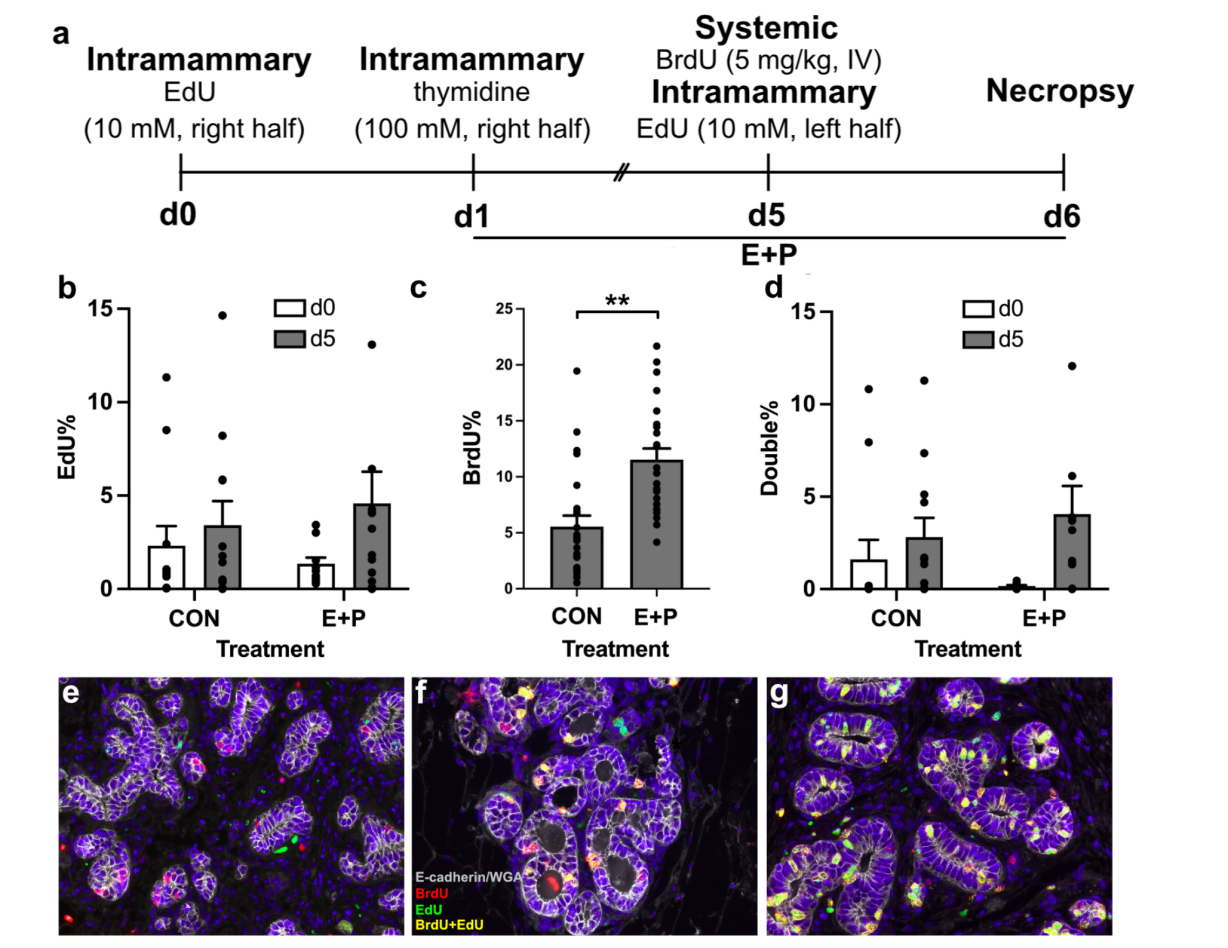
Intramammary labeling and hormone treatment. (**a**) At t 0, ethynyl deoxyuridine (EdU) was infused into the right udder half of all sheep, followed by thymidine 24 h later on d 1. Daily estrogen and progesterone (E + P) treatment of *n* = 3 ewes began on d 1 and continued until the last injection on d 5. Also on d 5, EdU was infused into the left udder half of all ewes, and bromodeoxyuridine (BrdU) was infused IV. Mammary tissues were collected at necropsy on d 6. (**b**) Intramammary EdU labeling index on d 0 and d 5 in control (CON) and E + P ewes differed over time (main effect, *p* = 0.05). (**c**) Labeling of MEC by systemic BrdU from d 5 was increased after treatment with E + P (*p* < 0.001). (**d**) Dual-labeled MEC were present in all glands, where their incidence differed by time (main effect *p* < 0.01, d 0 versus d 5), but not by treatment (CON versus E + P). (**e**) Representative image of epithelial structures in the mammary glands from necropsied sheep labeled at t 0 with intramammary EdU followed by unlabeled thymidine 24 h later, before E + P treatment commenced. Specimens were imaged to reveal cells that were positive for EdU (green), BrdU (red), E-cadherin and WGA (grey), and DAPI (blue). Scale bar equals 50 μm. (**f**) Labeling on d 5 after concurrent intramammary EdU and systemic BrdU delivered to a CON ewe. (**g**) Labeling on d 5 after concurrent intramammary EdU and systemic BrdU delivered to a ewe treated with E + P for 5 d

We next tested whether intramammary labeling could be used to detect increased levels of MEC proliferation in *n* = 6 ewes (8 mo old) in response to hormonal stimulation, as occurs during different stages of development. At the same time, we also sought to label rounds of MEC division at two specific timepoints using the distinct nucleotides, EdU and BrdU. At t 0 we infused 10 mM EdU into the right udder half of all ewes to capture a baseline level of proliferation (Fig. 2a). To delineate a 24 h labeling period, we next infused excess free thymidine (100mM, Sigma Aldrich, St. Louis, MO) into the right half of the udder to terminate EdU labeling. At the same time, we began treating *n* = 3 ewes with 17β-estradiol and progesterone (E + P, Sigma Aldrich, St. Louis, MO; 0.1 mg/kg and 0.25 mg/kg BW in ethanol, SC) for a total of 5 d. Three ewes remained untreated and served as controls (CON). On the last day of hormone treatment (d 5), EdU (10 mM) was also infused into the left udder half of all sheep. To allow for a direct comparison of intramammary and systemic labeling, we also infused BrdU (IV, 5 mg/kg BW) concurrently with the delivery of intramammary EdU on d 5. All sheep were necropsied 24 h later and tissue specimens were collected from four distinct regions within each udder half (Supplementary Fig. 1).

### Immunohistochemistry

Tissue specimens were fixed overnight in 4% paraformaldehyde, dehydrated and embedded in paraffin before sectioning at 5 μm onto charged microscope slides. Rehydrated sections were permeabilized with Triton-X (0.3% in phosphate buffered saline, PBS) followed by antigen retrieval in Tris (10 mM) EDTA (1 mM) buffer (pH 9) at 96ºC. Ethynyl deoxyuridine was detected via a 30 min copper-catalyzed click reaction that conjugated Alexa Fluor 488 to all EdU (all reagents from Vector Labs, Newark, CA). Cross-reactivity between EdU and the BrdU antibody was suppressed via a second click reaction in the presence of azidomethyl phenyl sulfide (2mM; Sigma Aldrich, St. Louis, MO) [31]. Sections were then blocked with 1% BSA and incubated for 2 h with a rat monoclonal anti-BrdU antibody (RRID: AB_305426; 1:100; Abcam, Cambridge, UK) and wheat germ agglutinin conjugated to Alexa Fluor 594 (1:1200; Biotium, Fremont, CA) in 1% BSA. After washing with PBS-0.05% Tween20 (PBST), sections were incubated with an Alexa Fluor 647-conjugated donkey anti-rat IgG (RRID: AB_2340694; 1:500; Jackson Immunoresearch, West Grove, PA) in 1% BSA for 2 h. Sections were then incubated with a rabbit monoclonal anti-E-cadherin antibody (RRID: AB_2291471; 1:100; Cell Signaling Technology, Danvers, MA) overnight at 4º C. Lastly, sections were washed with PBST and incubated with Alexa Fluor 594-conjugated donkey anti-rabbit IgG (RRID: AB_2340621; 1:500; Jackson Immunoresearch) in 1% BSA for 1 h. Nuclei were counterstained with DAPI (1:1000; Roche, Indianapolis, IN) and sections were mounted in ProLong Diamond Antifade Mountant (Thermofisher, Waltham, MA).

### Image Acquisition and Analysis

Each tissue section from an individual specimen was subdivided into five preplanned regions, where each field was captured in monochrome using a 20 × (NA 0.50) objective on an Olympus BX51 microscope (Olympus Life Science, Waltham, MA) fitted with a QICAM Fast1394 camera (Teledyne Photometrics, Tucson, AZ). The sequence of steps to analyze a single field was directed by an internal macro in Fiji to ensure consistency during analysis and file handling [32]. First, after manual outlining of all epithelial structures defined by E-cadherin and WGA, the StarDist 2D plugin was used to detect and quantify all MEC nuclei within a single image [33]. An average of 396 MEC per field (range 142–742 MEC) was quantified per individual field in Experiment 1 and an average of 530 MEC per field was analyzed in Experiment 2 (range 172–1121 MEC). Next, all EdU, BrdU, or dual-positive nuclei were counted manually in each respective monochrome channel. Positivity was validated by two separate examiners who were blinded to treatment. Labeling index was defined as the percentage of nucleotide-positive cells as a proportion of total MEC within an image.

### Statistical Analysis

Analysis was performed using R (v4.3.1) and the packages lme4 (v1.1-34) and emmeans (v1.8.7). Data from Experiment 2 was analyzed as a mixed-effects model with sheep as the experimental unit to test for the main effects of hormone treatment (E + P or CON), time of infusion (d 0 or d 5), and their interactions. Significance was declared at *p* < 0.05.

## Results

We first sought to determine the efficacy of EdU labeling of MEC following intramammary infusion of different doses of EdU and when compared to systemic BrdU. There was a dose-dependent effect of EdU on MEC labeling, where the percentage of labeled MEC increased from 0% to 0.2% in response to 0.1 and 1.0 mM, respectively to a maximum of 3.0% after infusion of 10 mM EdU (Fig. 1b).

We also examined MEC proliferation rates after IV infusion of BrdU that was delivered 24 h after the EdU infusion (Fig. 1c). The average rate of BrdU labeling differed between the ewes (*p* < 0.001), perhaps unsurprising given we did not control for their stage of estrous. Given this between-animal variation, we therefore deemed it appropriate to also normalize the EdU labeling data against the corresponding BrdU labeling index (EdU/BrdU) for each given field. This strategy generated normalized labeling indices of 0.0002, 0.34, and 0.35 for the 0.1 mM, 1.0 mM, and 10 mM EdU doses, respectively (Fig. 1d). Notably, there was greater variation about the mean for the 1.0 mM dose (0.34 ± 0.12) than for the 10 mM dose (0.35 ± 0.07, Fig. 1d). A large part of this variation among the dose levels could be explained by the observation that particularly among the lower doses, there was a greater frequency of fields that were negative for EdU uptake, whereas the same fields showed consistent levels of labeling by BrdU when it was delivered 24 h later (Supplementary Table 1).

Lastly, we also recorded MEC that were dual-labeled with both EdU and BrdU, where the proportion of these MEC increased with the concentration of EdU delivered (Fig. 1d). Again, this increased incidence of dual-labeling was largely a function of a greater number of EdU-positive fields (Supplementary Table 1). From this experiment, we concluded that among the treatments tested, 0.5 mL of 10 mM EdU provided the greatest labeling of MEC in the glands of nulliparous ewes.

We next sought to determine if a pulse-chase approach could be used to determine whether intramammary EdU would detect hormone-induced changes in MEC proliferation over time. At t 0, intramammary labeling was performed in the right gland of all sheep, followed by a chase with unlabeled thymidine 24 h later, before any hormone treatment started. By the time of necropsy, this dosing strategy yielded an average labeling index of 2.9% across *n* = 6 sheep (Fig. 2b). Half the animals were then treated with E + P for 5 d. During the last 24 h of this hormone treatment period, prior to necropsy, we dosed EdU into the unlabeled left udder half of all sheep, while we also labeled animals with BrdU IV for the same period. By the time of necropsy, CON glands had a final EdU labeling index of 3.4%, while the E + P glands had an EdU labeling index of 4.6% (*p* = 0.05, Fig. 2b). The labeling index detected by BrdU was 5.6% in CON glands, and 12% in glands from ewes treated with E + P (*p* < 0.001, Fig. 2c). There was also variation between the number of fields labeled with EdU (on d 0 or d 5) and fields labeled with BrdU (on d 5). In this case, BrdU labeling was detected in nearly every field, while EdU labeling was detected on average in 75% of fields (Supplementary Table 2).

We also observed dual-labeled MEC in both udder halves from all ewes. There was a higher proportion of these dual-labeled cells in the udder halves that received EdU and BrdU concurrently on d 5 during the last 24 h of E + P, versus in the contralateral glands when EdU was infused on d 0 and were subsequently exposed to IV BrdU on d 5 (*p* < 0.01) (Fig. 2d). We did not observe differences in dual-labeled MEC between udder halves in either CON or E + P treated animals.

## Discussion

The ability to label and track dividing MEC in the mammary gland has been an important tool for investigating normal development, stem cells, and cancer [12, 13, 15, 17]. Here we describe how intramammary delivery of nucleotides such as EdU provides for an alternative, feasible and cost-effective labeling route that can detect altered levels of proliferation in response to hormonal stimulation.

There are several potential benefits of an intramammary labeling method such as the one proposed here. The approach should be useful in a range of animal models, from mice that can have intraductal instillation performed using a fine syringe for various endpoints [34], to the largest of species for which clinical therapeutic infusions are often commonplace [3, 13]. Of note, not all species have a single galactophore at the teat/nipple, which would also present the opportunity to label independent ductal networks that drain to each, such as in pigs (2 galactophores), rabbits (8–10), and cats (3–7). Intramammary labeling of MEC division also affords the possibility of labeling different mammary glands within an animal, particularly when combined with a chase of free nucleotide that halts the uptake of the labeling nucleotide [35]. Furthermore, with appropriate consideration of downstream detection methodologies, this approach offers the opportunity to label the same gland repeatedly with two or three different thymidine analogs, as has been done using systemic delivery to repeatedly label the mammary glands or other tissues using combinations of BrdU, EdU, chlorodeoxyuridine, or iododeoxyuridine [14, 36–38].

Another major advantage of this intramammary labeling approach relative to traditional systemic delivery, whether that be IP [11, 12], IM [39], or IV [3, 13], is the reduced amount of labeling nucleotide that is required. A smaller amount of material not only affords the potential for greater user safety given the carcinogenic potential of these agents, but also can appreciably reduce the cost given that the intramammary dose used here (0.5 ml, 10 mM) cost approximately $2 based on current prices, whereas the delivery of EdU systemically (at 5 mg/kg) to these same sheep would have cost > $300/animal. While BrdU has a lower purchase cost and could be a logical substitute for intramammary labeling, EdU detection via the click reaction also brings the added benefit of rapid localization in tissue samples and eliminates the need for antigen-retrieval that can be damaging or inconsistent in thick tissue specimens.

There are certain aspects of this intramammary labeling method that warrant further consideration and testing. While intramammary EdU and systemic BrdU both labeled MEC, intramammary EdU did not label as many MEC as systemic BrdU when they were co-administered in Experiment 2. There are several potential explanations for this outcome. First, there may have been different uptake and incorporation of the two nucleotides, where certain lines of in vitro and in vivo evidence suggest that BrdU is preferentially incorporated into DNA relative to EdU [35, 40, 41]. An appropriate combination of experiments to address these questions would be to assess the rates of labeling after intramammary infusion of equimolar amounts of EdU and BrdU, and to reverse the routes of delivery (EdU IV versus intramammary BrdU).

The alternative, and potentially more likely, explanation is that the overall availability of EdU to the mammary epithelium was suboptimal. Indeed, from the range-finding in Experiment 1, we found that 0.5 ml of 10 mM EdU gave the highest rate of labeling, albeit this dose was likely less than saturating based on the demonstration that some fields were negative for EdU uptake (despite subsequently being BrdU-positive). The 0.5 ml infusate volume was chosen to match the estimated volume of the parenchymal ductal network in sheep based on previous experience, including the presence of a partially developed cistern [42], and to minimize the chance for leakage due to excessive internal pressure, which we also addressed by sealing the teat end. Several considerations warrant further refinement and validation on this front. The extent to which EdU degrades over time in vivo likely differs across tissues, where its stability and degradation in the mammary glands remains untested. In the circulation of mice, EdU has a clearance rate from plasma of 1 h [43]. A parallel consideration is the prospect that EdU diffused and was cleared across the epithelial border, which we suggest is likely given we frequently recorded examples of labeled stromal cells, basal to the epithelial compartment. Whether the uptake of EdU by MEC occurs through the apical membrane or requires leakage into the interstitium through leaking tight junctions is unclear, and may impact the utility of this method in states such as lactation when tight junctions are sealed.

## Conclusion

We present a method to label MEC directly via intramammary infusions. After establishing an optimal dose for the purposes of our study, we demonstrate that while intramammary labeling with a fixed dose of 0.5 ml at 10 mM does not label as many cells as systemic labeling, this method can detect altered levels of proliferation. Further, this method allows for treatment of glands separately, or repeatedly, to temporally label cells in the mammary glands. Our new method offers a unique and cost-effective opportunity to study cellular dynamics in the mammary glands across a wide range of species.

## Electronic supplementary material

Below is the link to the electronic supplementary material.

Supplementary Material 1

## Data Availability

Data is provided within the manuscript or the supplementary information files.
