# Supplementary material for: Intramammary Labeling of Epithelial Cell Division
Source: J Mammary Gland Biol Neoplasia. 2024 Oct 16;29(1):17. doi: 10.1007/s10911-024-09570-4 (PMC11485144; doi:10.1007/s10911-024-09570-4)

**Supplemental Table 1.** **Frequency of EdU- and BrdU-labeled mammary tissue from ewes in Experiment 1 according to specimen sampled, and microscopic field.** Separate udder halves of ewes (n = 2) were administered different intramammary doses of EdU according to the timeline in Figure 1. Nine or ten specimens were collected per udder half, according to the diagram in Supplemental Figure 1. Specimens were sectioned, and five microscopic fields were imaged, prior to analysis. Means were calculated either using values for all specimens or fields, regardless of whether an observation count was zero or not, or as means across either specimens or fields only when EdU or BrdU were detected. Asterisk indicates that there was only one positive field.

|  | **EdU dose (mM)** | | | |
| --- | --- | --- | --- | --- |
|  | **0** | **0.1** | **1.0** | **10** |
| **Specimens** |  |  |  |  |
| Number EdU-positive | 0/10 | 0/10 | 5/10 | 8/9 |
| Number BrdU-positive | 10/10 | 10/10 | 10/10 | 9/9 |
| **Fields** |  |  |  |  |
| Number EdU-positive | 0/50 | 1/46 | 17/49 | 32/45 |
| Number BrdU-positive | 47/50 | 37/46 | 40/49 | 43/45 |
|  |  |  |  |  |
| **Average EdU (% of MEC)** |  |  |  |  |
| Averaged, all specimens | 0 | 0.02 ± 0.02 | 0.2 ± 0.1 | 4.2 ± 0.5 |
| Averaged, positive-only specimens | 0 | 0.02 ± 0.06 | 0.5 ± 0.1 | 3.3 ± 0.8 |
| Averaged, all fields | 0 | 0.01 ± 0.01 | 0.2 ± 0.1 | 3.0 ± 0.5 |
| Averaged, positive-only fields | 0 | 0.6 ± 0.0* | 0.7 ± 0.1 | 4.2 ± 0.5 |
|  |  |  |  |  |
| **Average BrdU (% of MEC)** |  |  |  |  |
| Averaged, all specimens | 3.5 ± 0.6 | 1.5 ± 0.5 | 1.1 ± 0.2 | 6.5 ± 1.1 |
| Averaged, positive-only specimens | 3.5 ± 0.6 | 1.5 ± 0.5 | 1.1 ± 0.2 | 6.5 ± 1.1 |
| Averaged, all fields | 3.5 ± 0.5 | 1.6 ± 0.3 | 1.1 ± 0.2 | 6.5 ± 0.7 |
| Averaged, positive-only fields | 3.7 ± 0.5 | 1.9 ± 0.4 | 1.3 ± 0.3 | 6.8 ± 0.7 |

**Supplemental Table 2. Frequency of EdU and BrdU labeling across specimens and fields in Experiment 2 according to specimen sampled, and microscopic field.** One udder half for ewes (n = 6) was administered EdU at time zero, according to the timeline in Figure 2. Half of the ewes (n = 3) were then treated with estrogen plus progesterone (E+P) for 5 d. A single bolus of BrdU was delivered IV 24 h prior to necropsy and an intramammary dose of BrdU was also administered to the contralateral udder half. Four specimens were collected per udder half according to the diagram in Supplemental Figure 1, prior to fixation and processing for histology. Five fields were imaged per specimen. In total, 60 fields were analyzed across n = 6 ewes. Mean labeling index was calculated across specimens or fields, regardless of whether an observation was zero or not. Mean labeling index was also calculated across specimens or fields only when EdU or BrdU was detected.

|  | **Control** | | **E+P** | |
| --- | --- | --- | --- | --- |
|  | **Day 0** | **Day 5** | **Day 0** | **Day 5** |
| **Specimens** |  |  |  |  |
| Number EdU-positive | 12/12 | 10/12 | 12/12 | 11/12 |
| Number BrdU-positive | 12/12 | 12/12 | 12/12 | 12/12 |
|  |  |  |  |  |
| **Fields** |  |  |  |  |
| Number EdU-positive | 46/60 | 42/60 | 48/60 | 46/60 |
| Number BrdU-positive | 60/60 | 59/60 | 60/60 | 60/60 |
|  |  |  |  |  |
| **Average EdU%** |  |  |  |  |
| Averaged, all specimens | 2.3 ± 1.1 | 3.4 ± 1.3 | 1.4 ± 0.3 | 4.6 ± 1.7 |
| Averaged, positive-only specimens | 2.3 ± 1.1 | 4.1 ± 1.5 | 1.4 ± 0.3 | 5.0 ± 1.8 |
| Averaged, all fields | 2.3 ± 0.5 | 3.4 ± 0.6 | 1.4 ± 0.3 | 4.6 ± 0.8 |
| Averaged, positive-only fields | 3.0 ± 0.7 | 4.9 ± 0.9 | 1.7 ± 0.3 | 6.0 ± 1.0 |
|  |  |  |  |  |
| **Average BrdU%** |  |  |  |  |
| Averaged, all specimens | 6.5 ± 1.7 | 4.6 ± 0.6 | 12.5 ± 0.8 | 10.6 ± 0.6 |
| Averaged, positive-only specimens | 6.5 ± 1.7 | 4.6 ± 0.6 | 12.5 ± 0.8 | 10.6 ± 0.6 |
| Averaged, all fields | 6.5 ± 0.8 | 4.6 ± 0.6 | 12.5 ± 0.8 | 10.6 ± 0.6 |
| Averaged, positive-only fields | 6.5 ± 0.8 | 4.6 ± 0.6 | 12.5 ± 0.8 | 10.6 ± 0.6 |

**Supplementary Figure 1: Tissue specimen sampling at necropsy.** a) Experiment 1 tissue specimens were taken from 10 regions within each bisected the udder half. Specimens were sampled from anterior (regions 1 and 10), posterior (regions 5 and 6), and proximal, medial, and distal from the teat (regions 2-4 and 7-9). b) Experiment 2 tissue specimens were sampled from anterior (1 and 4) and posterior (2 and 3) regions of each bisected udder half.

Supplementary Figure 1.


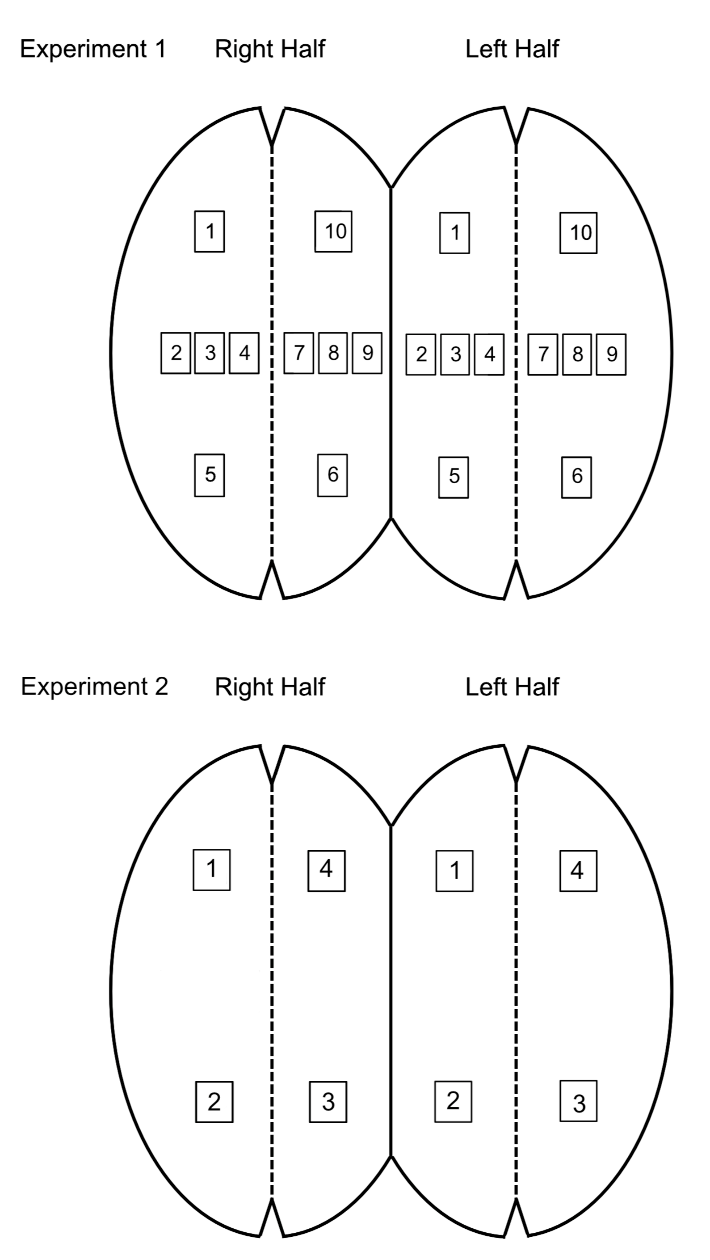

Supplement: Supplementary file 1 — Supplementary Material 1 [file 10911_2024_9570_MOESM1_ESM.docx]
